# Jewel Beetle Opsin Duplication and Divergence Is the Mechanism for Diverse Spectral Sensitivities

**DOI:** 10.1093/molbev/msad023

**Published:** 2023-01-31

**Authors:** Camilla R Sharkey, Jorge Blanco, Nathan P Lord, Trevor J Wardill

**Affiliations:** Department of Ecology, Evolution and Behavior, University of Minnesota, Saint Paul, MN; Department of Ecology, Evolution and Behavior, University of Minnesota, Saint Paul, MN; Department of Entomology, Louisiana State University Agricultural Center, Baton Rouge, LA; Department of Ecology, Evolution and Behavior, University of Minnesota, Saint Paul, MN

**Keywords:** Buprestidae, *Drosophila*, Coleoptera, Spectral tuning, Visual pigment, Insect vision

## Abstract

The evolutionary history of visual genes in Coleoptera differs from other well-studied insect orders, such as Lepidoptera and Diptera, as beetles have lost the widely conserved short-wavelength (SW) insect opsin gene that typically underpins sensitivity to blue light (∼440 nm). Duplications of the ancestral ultraviolet (UV) and long-wavelength (LW) opsins have occurred in many beetle lineages and have been proposed as an evolutionary route for expanded spectral sensitivity. The jewel beetles (Buprestidae) are a highly ecologically diverse and colorful family of beetles that use color cues for mate and host detection. In addition, there is evidence that buprestids have complex spectral sensitivity with up to five photoreceptor classes. Previous work suggested that opsin duplication and subfunctionalization of the two ancestral buprestid opsins, UV and LW, has expanded sensitivity to different regions of the light spectrum, but this has not yet been tested. We show that both duplications are likely unique to Buprestidae or the wider superfamily of Buprestoidea. To directly test photopigment sensitivity, we expressed buprestid opsins from two *Chrysochroa* species in *Drosophila melanogaster* and functionally characterized each photopigment type as UV- (356–357 nm), blue- (431–442 nm), green- (507–509 nm), and orange-sensitive (572–584 nm). As these novel opsin duplicates result in significantly shifted spectral sensitivities from the ancestral copies, we explored spectral tuning at four candidate sites using site-directed mutagenesis. This is the first study to directly test opsin spectral tuning mechanisms in the diverse and specious beetles.

## Introduction

Opsin proteins are G-protein-coupled transmembrane receptors. They have an essential role in animal photosensitivity and have also been shown to function in diverse nonvisual pathways (Porter et al. 2012; Leung and Montell 2017). Opsin proteins alone are insensitive to light but when coupled to a light-absorbing chromophore (derivative of vitamin A) they form a photosensitive unit (Terakita 2005). In the first steps of animal photoreception, the opsin and chromophore unit, termed photopigment or visual pigment, absorbs a single photon of light and a phototransduction signaling cascade is onset (Fain et al. 2010). Thus, the molecular basis of animal vision is underpinned by opsin proteins. The two major animal visual opsin gene classes—rhabdomeric-opsins (r-opsins) and ciliary-opsins (c-opsins)—are commonly the primary visual opsins of invertebrates and vertebrates, respectively (Porter et al. 2012). The ancestor of insects likely had three r-opsin genes (Briscoe and Chittka 2001). These form the three major opsin groups seen in extant insects: UV (ultraviolet-sensitive), SW (short-wavelength-sensitive), and LW (long-wavelength-sensitive) that broadly describe the spectral range of the photopigments they form.

The primary mechanism that expands the sensitivity of visual systems to different wavelengths of light is through duplication and diversification of the underlying opsin genes (van der Kooi et al. 2021). Spectral shifting of photopigments occurs through structural changes in the opsin protein that alters the environment of the light-absorbing chromophore (Terakita 2005). This process, termed spectral tuning, has been shown to occur within the chromophore binding pocket in both vertebrate and invertebrate photopigments (Yokoyama 2002; Wakakuwa et al. 2010; Liénard et al. 2021; Smedley et al. 2022). Predicting spectral shifts in photopigments requires experimental testing of proposed tuning sites through site-directed mutation. In vertebrate opsins, it has been shown that even a few structural changes within the binding pocket can lead to significant shifts in the sensitivity of the photopigment. One such well-known example, termed the “five-sites rule”, predicts that substitutions at just five sites can explain the spectral variation in green- and red-sensitive vertebrate photopigments (Yokoyama and Radlwimmer 1998). Three of these five sites induce spectral shifting through the loss or gain of a hydroxyl group. This particular structural change has been proposed as a major mechanism for spectral shifting in photopigments from diverse taxa, termed the “–OH” rule (Sekharan et al. 2012).

The structure of the chromophore itself also affects the spectral sensitivity of the photopigment. The adaptive function of chromophore switching has been demonstrated in the vertebrates as a mechanism for tuning the visual system to spectrally shifting environments (Corbo 2021). In insects, two major chromophore types are utilized: retinal (A1) and 3-hydroxyretinal (A3). A shortwave shift of 12 nm has been experimentally demonstrated in bovine rhodopsin bound to A3 rather than its native A1 (Gärtner et al. 1991), but the adaptive function of A1/A3 spectral shifts is not well studied in insects.

Opsin losses and duplications have shaped the molecular diversity of insect visual systems, reducing the range of wavelengths that can be detected and discriminated (Jackowska et al. 2007) or expanding sensitivities into new regions of the light spectrum (Liénard et al. 2021). The widely conserved SW opsin that underpins sensitivity to blue light has been lost in beetles (Sharkey et al. 2017), thereby reducing the capacity for complex color vision. However, there is growing evidence that some lineages may have expanded spectral sensitivities through UV and LW opsin duplications and subfunctionalization (Sharkey et al. 2017, 2021), but this has not yet been directly tested. Jewel beetles (Buprestidae) are a prime candidate to study photopigment sensitivity and spectral tuning in beetles. Buprestids are known to use color cues for both host (Poland et al. 2019) and mate detection (Gwynne and Rentz 1983; Domingue et al. 2016). In addition, they have been shown to possess complex spectral sensitivity with up to five spectrally distinct photoreceptor types ranging from UV- to red-sensitive (Meglič et al. 2020). Their opsin repertoire is also diverse due to two opsin duplication events resulting in four structurally divergent photopigment types: UV1, UV2, LW1, and LW2 (Lord et al. 2016).

We do not yet know the extent to which jewel beetle photopigments have spectrally diversified, as several other factors can influence the spectral sensitivity of insect visual systems. These include ocular pigments (e.g., screening or intrarhabdomal filters), self-screening and the type of chromophore, A1 (retinal) or A3 (3-hydroxyretinal) (Gleadall et al. 1989; Seki and Vogt 1998). Previous work has identified candidate sites for tuning in jewel beetle opsins using selection analyses and structural modeling (Lord et al. 2016; Sharkey et al. 2017), some of which are analogous to sites in other study animals that are known to influence spectral sensitivity. However, spectral tuning of opsins in beetles has yet to be verified in vivo, a necessary step in supporting hypotheses of shifted spectral sensitivities across Coleoptera.

Opsin expression systems enable direct testing of photopigments that are otherwise difficult to detect in species using ERG, such as the red-sensitive receptor in buprestids (Meglič et al. 2020) or pose a technical or logistical challenge for intracellular recordings. In contrast to vertebrates, spectral tuning of visual opsins has been directly tested in relatively few invertebrate species: butterflies (Wakakuwa et al. 2010; Frentiu et al. 2015; Liénard et al. 2021) and *Drosophila* (Salcedo et al. 2003, 2009). This is due to the difficulty in expressing functional invertebrate photopigment in cell culture (Knox et al. 2003). Prior to advancements in invertebrate cell culture methods (Liénard et al. 2022), heterologous expression of invertebrate opsin was achieved in *Drosophila*, allowing the characterization of membrane-bound *Limulus* and honeybee photopigments, in a photoreceptor environment (Townson et al. 1998; Knox et al. 2003). However, the *Drosophila* expression system has not yet been used to test site mutations in heterologous opsins. As more insect opsin data has become available for previously understudied groups (e.g., Odonata (Futahashi et al. 2015; Suvorov et al. 2017), Diptera (Feuda et al. 2021), Coleoptera (Sharkey et al. 2017, 2021)), we now have a large resource with which to explore and characterize photopigment sensitivity.

We have undertaken a study to characterize the spectral sensitivities of insect visual pigments using a *Drosophila* expression system. We characterized beetle opsins from the cowpea weevil (*Callosobruschus maculatus, Chrysomelidae: Bruchinae*) and two jewel beetle species, *Chrysochroa rajah* Gory and *Ch. mniszechii* Deyrolle (Buprestidae: Chrysochroinae). In addition, we included the monarch butterfly (*Danaus plexippus*) to show the utility of this system for future work in Lepidoptera, as a complementary method to cell culture. We expressed each opsin copy separately in an opsin-deactivated white-eye *Drosophila melanogaster* genetic background and characterized the in vivo functional spectral sensitivity using ERG. To verify the accuracy of our genetic and functional quantification methods for characterizing beetle opsins, we compared our results for cowpea weevil opsins with ERG measurements made in situ from adult beetles. We explored spectral tuning mechanisms in jewel beetles by characterizing four genetically engineered buprestid opsin variants by introducing point mutations. This was done to validate four best-candidate sites that would be predicted to induce spectral tuning, according to results from structural modeling.

## Results

### Opsin Evolution in Buprestidae

RNA-seq analysis yielded four full-length UV1, UV2, LW1, and LW2 opsins for each buprestid species examined: *Chrysochroa mniszechii*, *Ch. rajah*, *Agrilus zanthoxylumi*, *Capnodis tenebrionis* (Linnaeus), and *Ptosima undecimmaculata* (Herbst). Male and female *Cap. tenebrionis* samples yielded opsins with 100% sequence identity but with additional female UTRs in both UV copies, therefore only female opsins were retained for further analysis.

**Fig. 1.**
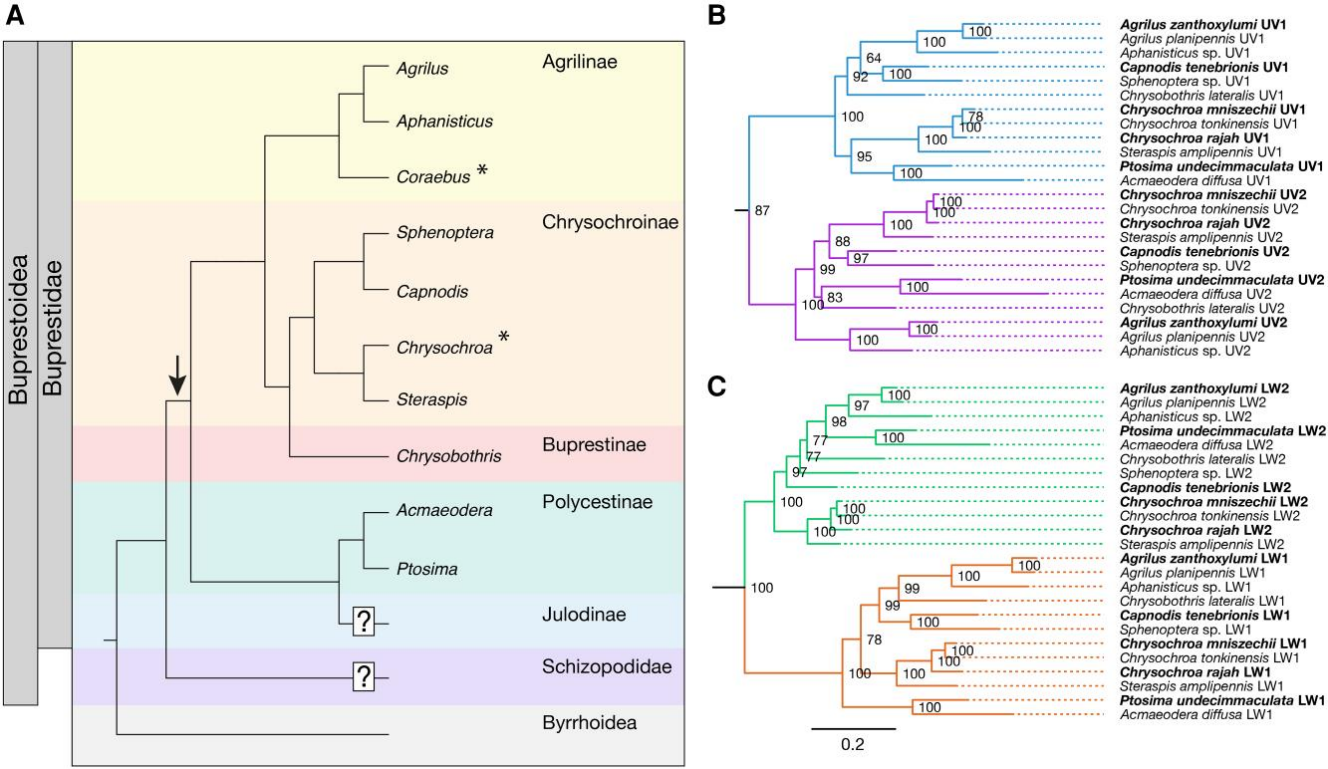
Opsin evolution in Buprestidae. (*A*) Species topology of the beetle family Buprestidae based on Evans et al. (2015) and Cai et al. (2022) showing all subfamilies, with the exception of Galbellinae, which is nested within Chrysochroinae and Buprestinae. All genera whose opsins have been previously described are shown: *Agrilus*, *Aphanisticus*, *Sphenoptera*, *Chrysochroa*, *Steraspis*, *Chrysobothris*, and *Acmaeodera*, as well as *Capnodis* and *Ptosima* (this study). *Coraebus* is also included as spectral sensitivity has been characterized (Meglič et al. 2020). The superfamily Byrrhoidea has been proposed as sister to superfamily Buprestoidea (Buprestidae + Schizopodidae) (McKenna et al. 2019; Cai et al. 2022). The proposed timings for UV and LW buprestid opsin duplication events are indicated by an arrow and subfamilies with no available opsin or spectral sensitivity data are indicated with a question mark. Asterisks indicate genera where spectral sensitivity have been characterized previously (*Coraebus* (Meglič et al. 2020)) and in this study (*Chrysochroa*). ML phylogenetic relationship of buprestid UV1 and UV2 opsins (*B*) and LW1 and LW2 opsins (*C*) New sequences from this study are indicated in bold. Node values are UFboot supports based on 10,000 replicates. See supplementary figure S1, Supplementary Material online for the full topology.

The recently proposed sister group to Buprestoidea (Buprestidae + Schizopodidae), Byrrhidae, alongside other putative sister taxa *Heterocerus fenestratus* (Thunberg) (Coleoptera: Byrrhoidea: Heteroceridae), and *Dryops* sp. (Coleoptera: Byrrhoidea: Dryopidae) (from a previous study (Sharkey et al. 2017)) were also examined, to determine the timing of buprestid UV and LW opsin duplication events. Phylogenetic analysis suggests that duplicate opsins found in both Byrrhidae species (*Byrrhus pilula* (Linnaeus) and *Notolioon* sp.) and other members of Byrrhoidea (*H. fenestratus* and *Dryops* sp.) were not orthologs to buprestid opsins. This indicates that the UV and LW opsin duplication events seen in buprestids are restricted to the family Buprestidae (fig. 1) or the wider superfamily of Buprestoidea.

### Validation of Beetle Opsin Expression in Transgenic *Drosophila*

To measure spectral responses from beetle visual pigments, we generated transgenic *Drosophila* with several important features. Firstly, the response of native *Drosophila* photoreceptors was inhibited via the *norpA* mutation, encoding phospholipase C (PLC). Activity of the more numerous, untiered outer photoreceptors (R1–6) was restored by selective expression of PLC. Finally, beetle opsin was expressed in the outer receptors alongside a nonfunctional mutant *Drosophila* Rh1 (*ninaE^8^*).

**Fig. 2.**
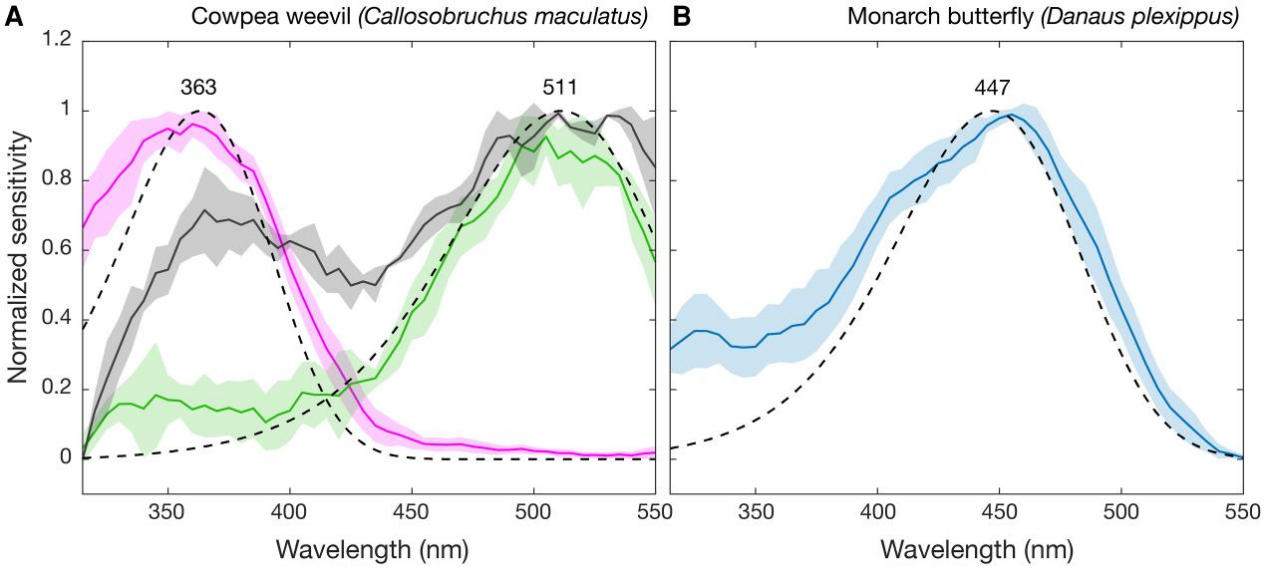
Spectral sensitivities of cowpea weevil and monarch butterfly visual pigments ectopically expressed in *Drosophila*. (*A*) Spectral response of transgenic *Drosophila* (*n* = 6) expressing the cowpea weevil *Callosobruchus maculatus* UV (363 nm, pink line) or LW (511 nm, green line) opsin with fitted visual pigment templates (Stavenga et al. 1993) (dashed lines) and predicted photopigment *λ*_max_. Spectral response of adult *C. maculatus* is shown in gray (*n* = 3). (*B*) Spectral response of transgenic *Drosophila* (*n* = 5) expressing monarch butterfly (*Danaus plexippus*) SW opsin with fitted visual pigment template (Stavenga et al. 1993) (dashed line) and predicted photopigment *λ*_max_. Error shown is standard deviation. See supplementary dataset S1, Supplementary Material online for sensitivity data and fitted templates.

To confirm that transgenic *Drosophila* could be used as a tool to successfully characterize beetle opsins, we tested the response of ectopically expressed UV and LW cowpea weevil (*Callosobruchus maculatus*) opsins (*n* = 6). For comparison, we also measured from lab-reared adults directly using ERG (*n* = 3) (fig. 2). *C. maculatus* was found to have only two opsin copies, one UV and one LW opsin, both of which were functional in *Drosophila*, allowing for spectral quantification. Spectral sensitivities of ectopically expressed UV and LW photopigments in *Drosophila* closely approximated the UV- and green-sensitive photoreceptor responses, measured from adult *C. maculatus*, using ERG (fig. 2*A*). Furthermore, to demonstrate the utility of *Drosophila* opsin expression systems for future studies, we expressed the lepidopteran SW opsin from the monarch butterfly (*Da. plexippus*) and successfully characterized spectral response of this photopigment (fig. 2*B*).

### Spectral Response of *Chrysochroa* Visual Pigments

**Fig. 3.**
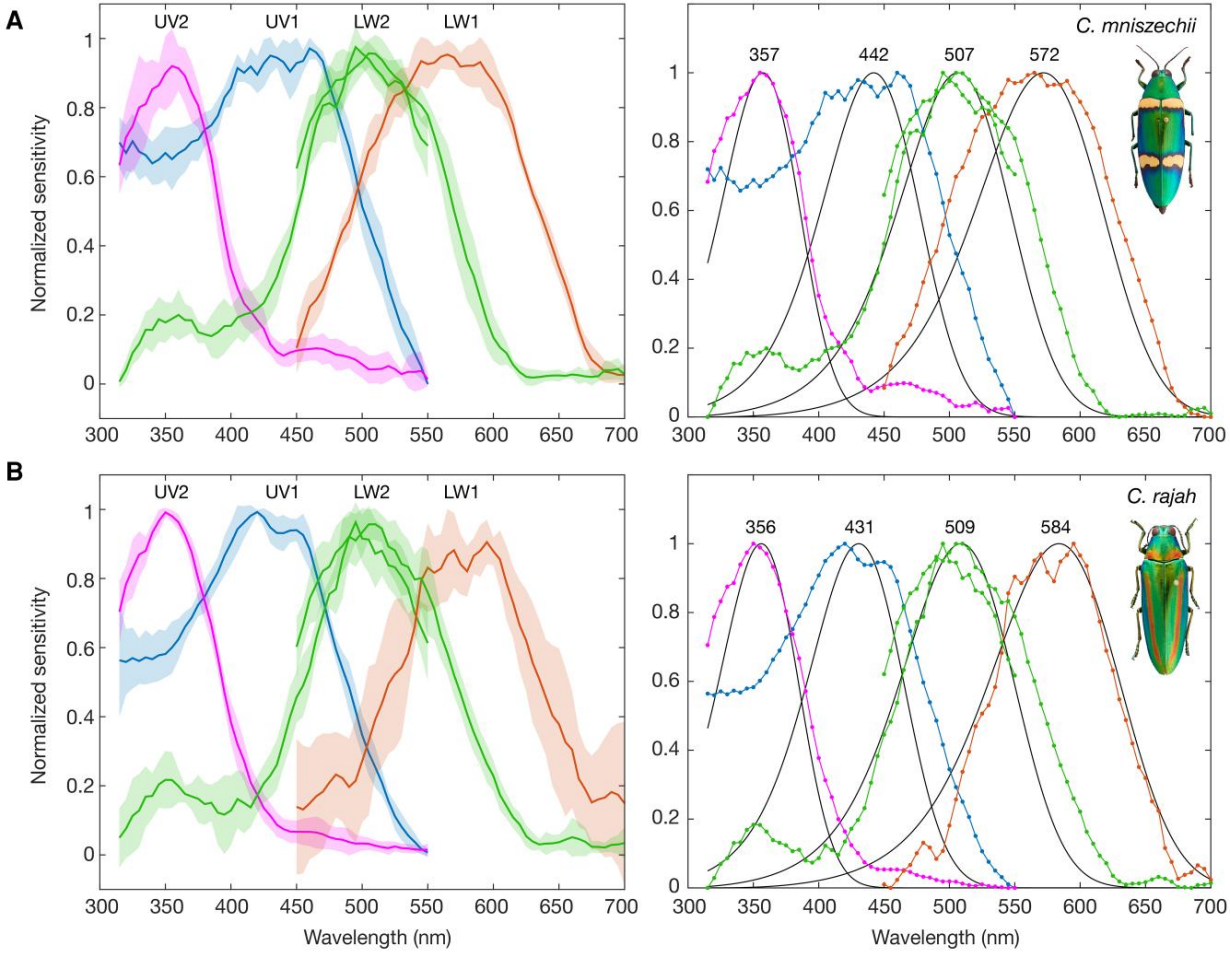
Spectral sensitivities of *Chrysochroa* visual pigments ectopically expressed in *Drosophila*. Photoreceptor response of transgenic *Drosophila* expressing *Chrysochroa mniszechii* (*A*) and *Ch. rajah* (*B*) UV1, UV2, LW1, or LW2 opsins. Mean response and standard deviation (*n* = 6) (left) and the mean responses fitted with a visual pigment template (right) (Stavenga et al. 1993). The estimated *λ*_max_ for each visual pigment is shown (nm). Prior to visual pigment template fitting, mean responses were renormalized between 0 and 1. Data for the LW2 opsin is shown for 315–550 nm and 450–700 nm and the curve was fit to the 315–550 nm testing range (see text). See supplementary dataset S1, Supplementary Material online for sensitivity data and fitted templates.

We expressed four buprestid opsin classes UV1, UV2, LW1, and LW2, from two species, *Ch. mniszechii* and *Ch. rajah*, in white-eye *Drosophila* and the resulting photopigment responses were characterized using ERG (*n* = 6 for each opsin). Our results show that each opsin class forms a photopigment with a distinct sensitivity range: UV-, blue-, green-, and orange-sensitive (fig. 3). All opsins were functional and the ERG waveform was typical for *Drosophila* outer photoreceptors where these opsins are expressed, with fast on and off transients flanking a sustained negative photoreceptor voltage response. Visual pigment templates fit well to UV2, LW1, and LW2 spectral sensitivity curves (adjusted *R*^2^ > 0.8; supplementary table S1, Supplementary Material online) but poorly estimated the blue-sensitive UV1 photopigment for either species (adjusted *R*^2^ < 0.2; supplementary table S1, Supplementary Material online). The *λ*_max_ of photopigments were estimated by template fitting (Stavenga et al. 1993) for *Ch. mniszechii* at 357 nm (UV2), 442 nm (UV1), 507 nm (LW2), and 572 nm (LW1) and for *Ch. rajah*, at 356 nm (UV2), 431 nm (UV1), 509 nm (LW2), and 584 nm (LW1). Fitted curve *λ*_max_ estimates for LW2 in the two experimental testing regions, 315–550 nm/450–700 nm, differed by 8 nm in *Ch. mniszechii* and 7 nm in *Ch. rajah* (supplementary table S1, Supplementary Material online). The fit to the shorter testing region was therefore used for equivalent comparison with LW2 mutants (see below).

### Spectral Tuning in *Chrysochroa*

**Fig. 4.**
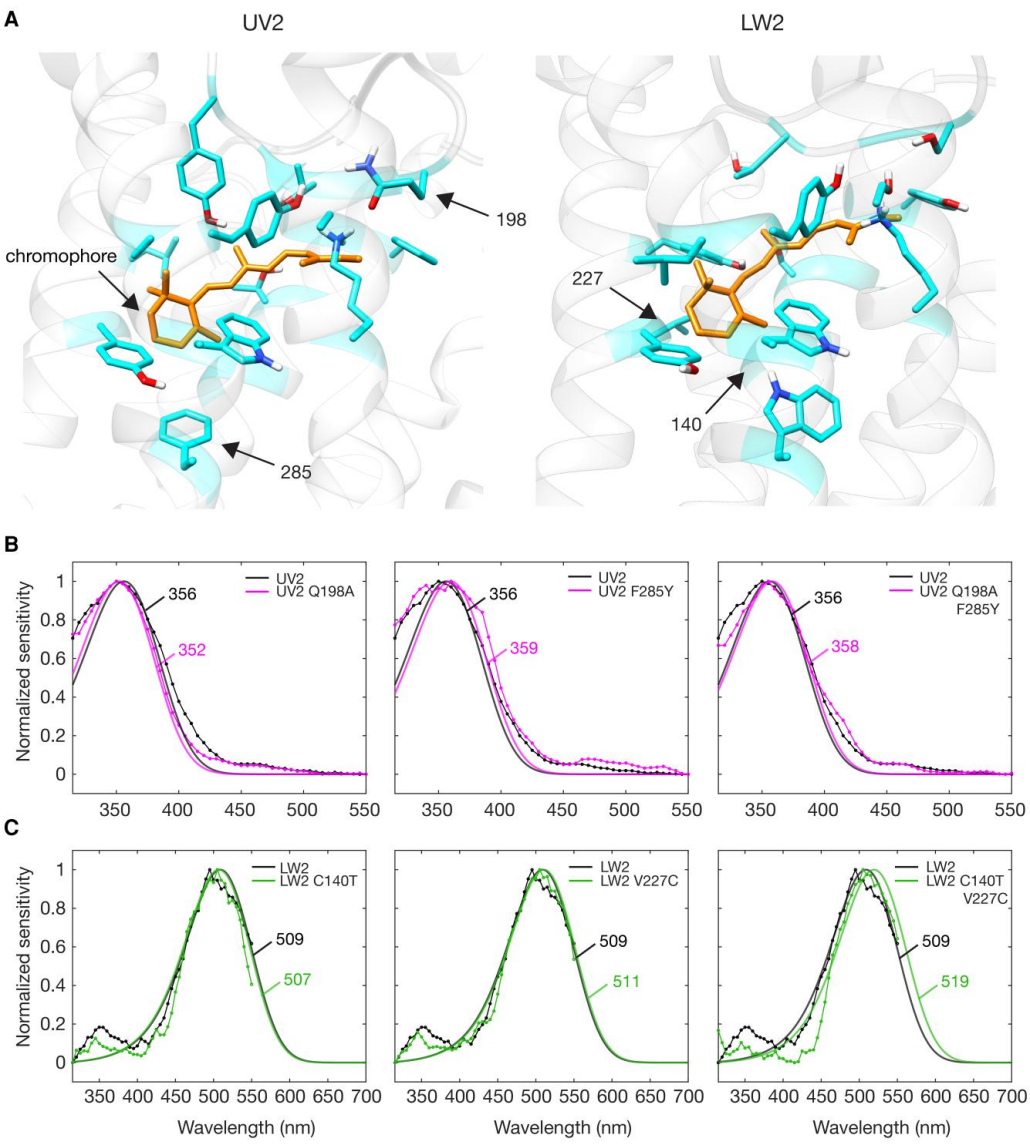
Spectral sensitivities of *Chrysochroa rajah* mutant photopigments. (*A*) 3D modeling of *Ch. rajah* UV2 and LW2 opsins highlighting all sites within the binding pocket surrounding the chromophore. Arrows indicate the sites mutated in this study and the chromophore in *A*. (*B*) Spectral sensitivities of *Drosophila* expressing wild-type UV2 opsin (*n* = 6), opsin with single mutations Q198A, F285Y and opsin with both mutations (*n* = 4). (*C*) Spectral sensitivities of *Drosophila* expressing wild-type LW2 opsin (*n* = 6) and opsin with single mutations C140T, V227C and opsin with both mutations (*n* = 4). Visual pigment templates (Stavenga et al. 1993) were fitted to wild-type UV2 and LW2 spectral responses in the equivalent wavelength testing range to mutant opsins (315–550 nm). The *λ*_max_ of fitted templates is also shown (nm). See supplementary dataset S1, Supplementary Material online for sensitivity data and fitted templates.

The four best-candidate tuning sites for UV and LW opsins were chosen according to the following criteria: their proximity to the chromophore (fig. 4*A*) and variation between opsin duplicates with the potential to exert structural change within the chromophore binding pocket (table 1). In total, 18 sites were predicted to be within the chromophore binding pocket in UV2 and 17 sites in LW2, with 16 sites shared between them. Of these, only five UV2 sites and three LW2 sites were variant between both *Ch. rajah* and *Ch. mniszechii* opsin duplicates (table 1 and supplementary tables S2 and S3, Supplementary Material online). All sites are numbered according to *Ch. rajah* UV2 or LW2 unless indicated otherwise. UV sites 130 and 217 were variant between *Chrysochroa* opsin duplicates but were predicted to have less significant structural change than the chosen candidate sites. Site 316 was only variant at this site within *Chrysochroa* (table 1 and supplementary table S2, Supplementary Material online). Two LW site substitutions did have the potential to exert structural change but were invariant in all but *Ch. rajah*, 204 or only variant within *Chrysochroa*, 298 (supplementary table S3, Supplementary Material online). Expanding site selection to include all sites within 5Å of the chromophore yielded only three additional variant sites between *Chrysochroa* orthologs (UV2 site 126, LW2 sites 132 and 224) but substitutions were not predicted to be structurally significant (glycine/alanine and serine/threonine).

**Table 1.** Candidate Spectral Tuning Sites.

Opsin	Site (bovine)	*Site (Ch. rajah)*	Copy 1	Copy 2	Predicted structural change
UV	118	130	S	T	Most likely insignificant
**UV**	**186**	**198**	**A**	**Q**	**Change in polarity and size**
UV	207	217	I	L	Most likely insignificant
**UV**	**261**	**285**	**Y**	**F**	**Change in polarity and hydroxyl group**
UV	292	316	S	A	Change in polarity
**LW**	**122**	**140**	**T**	**C**	**Change in polarity and hydroxyl group**
**LW**	**211**	**227**	**C**	**V**	**Change in disulfide bonding**

Sites predicted to be in the chromophore binding pocket of either UV2 or LW2 *Ch. rajah* opsins, variant between *Ch. rajah* and *Ch. mniszechii* opsin duplicates but invariant between species. Sites are numbered according to bovine rhodopsin and *Ch. rajah* UV2 or LW2 opsin. Each site was examined for predicted structural change (see Lord et al. 2016) and how variant residues were across the nine non-chrysochroines (see text). Based on these criteria, four candidate sites (in bold) were chosen to test for spectral shifts between opsin duplicates in both opsin classes: UV1/UV2 (two sites) and LW1/LW2 (two sites).

UV2 sites 198, 285 and LW2 sites 140, 227 were chosen for site-directed mutagenesis (table 1, bolded). All four single mutant opsins (one site mutation) and two double mutant opsins (two site mutations) were functional within the *Drosophila* expression system (*n* = 4 for each mutant opsin). However, our results indicated only marginal shifting in peak sensitivity in response to site mutation (fig. 4*B*and*C*; supplementary fig. S2, Supplementary Material online). Fitting pigment templates (Stavenga et al. 1993) estimated UV2 mutations to give rise to only a −4 nm shift (Q198A) and a +3 nm shift (F285Y) from the wild-type buprestid UV2 opsin. UV2 double mutants had a *λ*_max_ of only +2 nm from wild-type (fig. 4*B* and supplementary table S1, Supplementary Material online). Similarly, mutations to LW2 shifted the predicted *λ*_max_ by only −2 nm (C140T) and +2 nm (V227C) from the wild-type buprestid LW2 opsin. When both mutations were introduced, *λ*_max_ was shown to shift by +10 nm from wild-type (fig. 4*C* and supplementary table S1, Supplementary Material online). Mutant LW2 opsin *λ*_max_ was compared with measurements from wild-type LW2 using the equivalent experimental testing range (315–550 nm).

## Discussion

### Evolution of Expanded Wavelength Sensitivity in Buprestids

Our results show that each *Chrysochroa* (subfamily: Chrysochroinae) opsin group, UV1, UV2, LW1, and LW2, forms a photopigment with a distinct spectral profile, with each *λ*_max_ distributed evenly across a range from approximately 360 to 580 nm. Orthologs of all four opsin classes: UV1, UV2, LW1, and LW2 have been found in all buprestid species examined to date, across four of six subfamilies (Lord et al. 2016). This sampling spans the diversity of Buprestidae with the exception of subfamily Julodinae, which has been placed within the most “primitive” lineage of Buprestidae alongside *Acmaeodera* and *Ptosima*, and the enigmatic subfamily Galbellinae, which is nested within Buprestinae and Chrysochroinae (Evans et al. 2015). Orthologs of buprestid opsins were not recovered in *B. pilula*, *Notolioon* sp., or *H. fenestratus*, nor the previously recovered *Dryops* sp. (Sharkey et al. 2017). These taxa are members of the coleopteran superfamily Byrrhoidea, which is currently recognized as the sister group to the Buprestoidea (Buprestidae + Schizopodidae) (McKenna et al. 2019; Cai et al. 2022). Our results suggest that the UV and LW duplications are unique to Buprestidae or Buprestoidea (family Schizopodidae has not been analyzed to date), occurring early in the evolutionary history of this lineage, likely after the proposed split from Byrrhoidea ∼165 to 235 MYA (Cai et al. 2022).

The spectral sensitivity of buprestid species *Coraebus undatus* has been well characterized using intracellular recordings (Meglič et al. 2020) and therefore can now be compared with the photopigment spectral recordings in this study. Each *Chrysochroa* opsin duplicate forms a visual pigment with a unique spectral sensitivity, with UV duplicates UV1 and UV2 forming blue and UV-sensitive photopigments and LW duplicates LW1 and LW2 forming red/orange and green-sensitive photopigments, respectively (fig. 3). Intracellular measurements from *Cor. undatus* in subfamily Agrilinae revealed blue (430 nm), green (540 nm), and red (600 nm) photoreceptors (Meglič et al. 2020). In addition, two UV photoreceptor types were described, one peaking at 350 nm and the other, less commonly encountered, at 335 nm (Meglič et al. 2020). Our results from *Chrysochroa* suggest that it is likely that the additional UV photoreceptor type originates from screening of the single UV-sensitive photopigment. Both the major UV photoreceptor (350 nm) and blue photoreceptor (430 nm) align broadly with our measured values for UV2 and UV1 buprestid photopigments at 356–357 nm and 431–442 nm, respectively. It was suggested by Meglič et al. that the red-receptors of *Coraebus* (*λ*_max_ 600 nm) are LW shifted due to screening, owing to its steep spectral profile. Our results are in line with this suggestion with the *Chrysochroa* LW1 photopigment peaking at 572–584 nm. Our measurements for the green-sensitive LW2 photopigment point to a shorter *λ*_max_ in *Chrysochroa* (507–509 nm) than *Coraebus* (540 nm) possibly indicating interspecies variation in this photopigment sensitivity.

Characterizing the spectral profile of photopigments outside of their native environment allows us to determine the underlying sensitivities of such pigments and crucially, enables testing of hypotheses regarding the structure and function of insect opsins. In vitro methods for expressing and characterizing wild-type and mutant insect opsins using cell culture have been developed for use with a number of different invertebrate taxa (see review Liénard et al. 2022). We propose the *Drosophila* expression system as a complementary alternative to these systems and show its utility for characterizing beetle and lepidopteran opsins. The *Drosophila* expression system, in contrast to cell culture, allows us to maintain the membrane-bound opsin in a photoreceptor environment.

Our results from *C. maculatus* establish that UV and LW beetle opsins expressed in *Drosophila* closely approximate beetle photoreceptor sensitivities (fig. 2). This suggests that the native nonfunctional *Drosophila* Rh1 visual pigment and UV-sensitizing pigment did not strongly affect the beetle photopigment spectral profile. However, we cannot discount the possibility that spectral shifts are induced by interactions with *Drosophila* ocular pigments. Beetle opsin was expressed in untiered *Drosophila* outer receptors, in white eyed flies, thereby reducing the filtering effects of self-screening and screening pigment that can shift photopigment sensitivity (Sharkey et al. 2020). The chromophore utilized by *Drosophila* and thus coupled to the expressed beetle opsins in this study, 3-hydroxyretinal (A3), is likely not the native chromophore in *C. maculatus*, as beetles primarily use retinal (A1) chromophore (Gleadall et al. 1989). It is not yet known what effect chromophore switching has on insect opsins but according to vertebrate data, we might expect a shortwave shift in photopigments bound to A3 (Gärtner et al. 1991).

The spectral sensitivity of *C. maculatus* is relatively simple, with only two photoreceptor types, UV- and green-sensitive. We might expect to see spectral shifting effects of screening (ocular pigments and self-screening by distal photoreceptors) to be pronounced in the photoreceptors of beetle species with more spectrally complex retinas and tiered photoreceptors, such as those in buprestids (Meglič et al. 2020). Despite this, our measurements from a distantly related genus, *Chrysochroa*, aligns broadly to that of *Coraebus*, a species that has been well characterized using intracellular recordings (Meglič et al. 2020). We cannot discount the possibility that there is large spectral variation within opsin spectral classes across different buprestid species. However, we propose that the UV2, UV1, LW2, and LW1 opsins of buprestidae broadly align to UV-, blue-, green-, and LW-senstivity, according to the conservation of these genes across Buprestidae, our measurements and the intracellular recordings of distantly related *Coraebus*.

### Spectral Tuning Between *Chrysochroa* Opsin Orthologs

The finding that novel blue- and orange-sensitive photopigments have indeed evolved from the ancestral coleopteran UV- and green-sensitive photopigments (Sharkey et al. 2017) provides a useful model for testing potential spectral tuning sites in beetles. Buprestidae opsins have been well sampled across their diversity and therefore the variation at chromophore binding sites between and within opsin classes can now be evaluated and tested. *Chrysochroa* opsin spectral sensitivity differed between the two study species. Although both UV (UV2) and green (LW2) responses were near identical between the two species, *Ch. rajah* and *Ch. mniszechii*, variation was seen in the blue (UV1 *λ*_max_: 431 and 442 nm) and orange-sensitive photopigments (LW1 *λ*_max_: 584 and 572 nm).

*Chrysochroa rajah* and *Ch. mniszechii* UV1 opsin proteins share 95% sequence identity and all predicted sites within the chromophore binding pocket were invariant. However, as the spectral responses of the UV1 photopigment did not fit well to a modeled visual pigment template (Stavenga et al. 1993), the predicted *λ*_max_ should be interpreted cautiously. LW1 duplicates fit well to a visual pigment template (Stavenga et al. 1993) and are therefore more compelling for making such inferences. *Ch. rajah* and *Ch. mniszechii* LW1 opsin proteins share 86% sequence identity and one substitution was found within the binding pocket, at site 298. The substitution observed between *Ch. rajah* and *Ch. mniszechii* LW1 opsins (T298A) has the potential to affect the chromophore due to a loss of polarity and –OH group at the ionone ring (Sekharan et al. 2012). This site is a major tuning site in vertebrate green and red photopigments, one of the five site substitutions that describe the spectral variation in M/LW photopigments (Yokoyama and Radlwimmer 1998). We would predict a short-wavelength shift for a T298A substitution, according to mutagenesis studies in vertebrates (Neitz et al. 1991; Chan et al. 1992; Asenjo et al. 1994; Sun et al. 1997). The magnitude of this shift was observed to be in the range of 10–16 nm in vertebrate opsin, but it is difficult to make such predictions for beetle opsins, due to the structural differences between these distantly related opsins and difference in chromophore structure. The 12 nm short-wavelength shift observed in *Ch. mniszechii* LW1 photopigment may be attributed to this single site mutation but direct testing would be necessary to confirm this.

### Spectral Tuning Sites Between Buprestid Opsin Classes

The shifts in sensitivity of *Chrysochroa* photopigment types: from UV (∼360 nm) to blue (∼430–440 nm) and green (∼510–515 nm) to orange (∼570–580 nm) are considerable. Based on vertebrate and invertebrate studies of spectral tuning, we expect to find structural changes at the chromophore to reflect these shifts. *Ch. rajah* UV and LW opsin duplicates share only 73% and 68% identity, respectively, but there are relatively few variant sites in the chromophore binding pocket. Using structural modeling, we predicted the chromophore binding pocket to include 18 sites in UV2 and 17 in LW2 opsins. However, many of these were not variable between opsin duplicates (i.e., UV1 vs. UV2 or LW1 vs. LW2) in our study species. Of the six variant UV sites identified, three had substitutions that were predicted to exert little structural change at the binding pocket: threonine to serine and leucine to isoleucine. UV site A316S has the potential to alter the chromophore environment through a change in polarity; however, this site was only variable within *Chrysochroa* and invariant in other buprestids. As we were interested in potential core mutation sites in buprestid duplicate opsins, this site was not tested in this study. In addition, the same substitution from alanine to serine (bovine A292S) has been implicated in short-wavelength shifting in vertebrates (Sun et al. 1997; Takahashi and Ebrey 2003) and *Drosophila* (Salcedo et al. 2009) rather than LW shifting, which might predict a UV to blue-wavelength shift. Similarly, the third untested LW2 candidate spectral tuning site, 298, was invariant in all but one non-chrysochroine opsin.

None of the four best-candidate spectral tuning sites strongly influenced the spectral response of the UV or LW photopigments when tested as single mutations. LW2 double mutants, however, did exhibit a shift of +10 nm from wild-type, suggesting an additive and amplifying effect of additional mutations. This finding, however, does not describe the full 75 nm LW shift from LW2 to LW1 opsins we observe in *Ch. rajah*. Furthermore, we were unable to induce any meaningful shifting in the UV2 photopigment. Thus, more work is needed to examine other regions of the opsin for structural change such as those near the counterion, at bovine site 181 (Terakita et al. 2004). The counterion is responsible for stabilizing the Schiff base, the attachment point of the chromophore to the opsin protein and changes in the environment surrounding the Schiff base has been shown to induce shifting in vertebrate opsins (Hunt et al. 2001). The mechanism underlying spectral shifting between buprestid opsin duplicates is therefore unresolved and requires further investigation.

Insects vary in chromophore use across the order and even within single taxa in different regions of the eye (Seki et al. 1989). *Drosophila* and other flies in the group Cyclorrhapha are unique in producing the 3-S enantiomer of 3-hydroxyretinal (A3-S), while all other insects utilize either retinal (A1) or the 3-R enantiomer of 3-hydroxyretinal (A3-R) (Seki et al. 1994; Seki and Vogt 1998). A1 has been detected in one species of buprestid (*Chrysobothris*) (Smith and Goldsmith 1990). Therefore, similar to cell culture studies of butterfly opsins which use non-native A1 retinal (Wakakuwa et al. 2010; Liénard et al. 2021, 2022), the buprestid opsins in this study were bound to the non-native A3 chromophore.

Models of spectral shifting have been well established for vertebrates and allow predictions of *λ*_max_ by examining the interactions between opsin residues and retinal chromophore. One such model predicts that hydroxyl (–OH) groups act to stabilize retinal when in proximity to the b-ionone ring, resulting in a LW shift (Sekharan et al. 2012). In this study, we would predict UV2 F285Y and C140T might induce such effects as –OH groups are introduced by these mutations, but we did not observe this effect here. It may be possible that due to the structural differences in A1 and A3 chromophore, spectral shifting mechanisms differ between opsins bound to A1 retinal and A3 3-hydroxyretinal. We have shown here that this *Drosophila* expression system shows promise for lepidopteran opsin characterization and thus may be a complementary method that allows spectral testing using native A3. Further work is needed to determine if the action of spectral shifting mechanisms is chromophore-specific in insects (Gleadall et al. 1989).

## Materials and Methods

### Beetle Opsin Extraction

Total RNA was extracted from two beetle species (*Chrysochroa rajah* and *Ch*. *mniszechii*) using NucleoSpin RNA II isolation extraction kids (Clontech) and reverse-transcribed into cDNA libraries using the Illumina TruSeq RNA v2 sample preparation kit. The prepared mRNA libraries were sequenced on an Illumina HiSeq 2000 utilizing 101-cycle paired-end reads by the Microarray and Genomic Analysis Core Facility at the Huntsman Cancer Institute at the University of Utah (Salt Lake City, UT, USA). Transcriptomes were trimmed of adapter sequences and poor quality bases using Trimmomatic (Bolger et al. 2014) and assembled using Trinity (v 2.1.1) with trimming settings: SLIDINGWINDOW:4:5 LEADING:5 TRAILING:5 MINLEN:25. Available RNA-seq data from the Sequence Read Archive (SRA) for the cowpea weevil, *C. maculatus,* were combined per sex and assembled as described above. In addition, RNA-seq data from three buprestid species (*Capnodis tenebrionis*, *Agrilus zanthoxylumi*, and *Ptosima undecimmaculata*), two putative sister taxa from Byrrhidae (*B. pilula* and *Notolioon* sp.) and an additional Byrrhoidea species, *H. fenestratus* were assembled using Trinity (v 2.11). See supplementary table S4, Supplementary Material online for all SRA accession numbers.

Opsin sequences were extracted from assembled transcriptomes by first predicting coding regions (TransDecoder v5.5.0) and the longest open reading frame retained (ORF). In addition, all ORFs were BLASTed (BLAST + v2.2.31 and v2.9.0) against orthodb database EOG8NKF98 plus Lampyridae and *Thermonectus marmoratus* opsins, with an e-value threshold of 0.001. A homology search between remaining ORFs and our opsin database was carried out using hmmscan in HMMER (v3.1 and 3.3) (Eddy 2011) and final opsin sequences retained. Opsins were verified using BLASTp (https://blast.ncbi.nlm.nih.gov/) and by phylogenetic analysis with known insect opsins (see supplementary dataset S2, Supplementary Material online for sequence accession numbers). Sequences were also manually verified by alignment with beetle opsins from this dataset. Pseudogenes, non R-opsins, contaminant sequences and opsins with > 99% amino acid similarity were removed (CD-hit v4.8.1) (Li and Godzik 2006; Fu et al. 2012). A fragment of length 197 amino acids was obtained from *Agrilus zanthoxylumi*. As the sequencing was performed on head tissue therefore likely yielding all functional opsins, this fragment was not included in further analysis and possibly reflects a pseudogene. Similarly, the additional fragment from *Agrilus planipennis* named LW3 opsin was not included as this did not cluster with other buprestid LW opsins and is not known if it is functional. Opsin sequences in this study were aligned (MAFFT v7.453) (Katoh and Standley 2013) with additional insect opsins and cephalopod outgroup opsin (see supplementary dataset S2, Supplementary Material online). Phylogenetic analysis was performed on DNA sequences using codon-alignment in IQ-TREE (v 1.6.12) (Nguyen et al. 2015) and the substitution model SYM + I + G4 was selected automatically using ModelFinder (Kalyaanamoorthy et al. 2017).

### Transgenic *Drosophila*

Total RNA was extracted from male *C. maculatus* (Gen Elute, Sigma-Aldrich) and used as a template to synthesize the cDNA (verso cDNA synthesis kit, ThermoScientific). For *Chrysochroa* spp., the cDNA was available from previous sequencing steps (see above). Adult monarch butterfly (*Da. plexippus*) mRNA was provided as a gift from Emily Snell-Rood (University of Minnesota) and used to synthesis cDNA (verso cDNA synthesis kit, ThermoScientific). Monarch SW opsin (accession: AY605544), *C. maculatus* opsins (UV and LW) and *C. rajah* and *Ch. mniszechii* opsins (UV1, UV2, LW1, and LW2) were PCR-amplified from cDNA using Phusion High-Fidelity (New England BioLabs) or CloneAmp HiFi (Takara) DNA polymerase and species-specific opsin variant primer combinations (see supplementary table S5, Supplementary Material online). Overhang regions were introduced at the start (21 bp) and end (16 bp) of the sequences and the bovine epitope 1D4 (TETSQVAPA) was introduced to the end of each opsin, before the stop codon.

The PCR-amplified opsins were extracted from gels and purified using standard protocols (Nucleospin Gel and PCR Clean-up, Macherey-Nagel). Each opsin was then inserted into the pigActGFP vector (Sharkey et al. 2020), downstream of the *Drosophila melanogaster* Rh1 (*ninaE*) promotor, using recombination cloning (In-Fusion, Takara). A modified version of pigActGFP, named pActEHG-attB, was also used for the opsin cloning step. This vector contains the attB sequence, which allows for the phiC31 integrase-mediated insertion of DNA constructs at specific attP docking sites present in the *Drosophila* genome. This vector, with the insertion site attP-3B (VK000001), was used for monarch butterfly SW opsin and trialed in beetles using *Ch. mniszechii* UV1. As we observed lower amplitude ERG responses in transgenic flies expressing beetle opsins using this vector, it was not used for the remaining beetle opsins in this study.

All final plasmid constructs were purified using the NucleoBond PC100 kit (Macherey-Nagel) and submitted to Sanger sequencing before injecting into *Drosophila* embryos following standard protocols. The pigActGFP-opsin constructs (supplemented with the piggyBac helper plasmid) were injected into *PBac{actin88F > RFP, Rh1 > norpA}* embryos (Sharkey et al. 2020). The *pActEHG-attB* derived construct was injected into y(1)w[*]vas-int; PBac{actin88F > RFP, Rh1 > norpA ([*y+*]*attP-3B*)*VK00001* embryos. Transgenic flies containing the beetle opsin constructs inserted on the second chromosome were selected and loss-of-function mutations in *norpA* and *ninaE* were introduced by fly crossings to generate the following genotype: *w(1) norpA[36]; PBac{actin88F > RFP, Rh1 > norpA}, PBac{actin88F > GFP, Rh1 > Beetle opsin}; ninaE(8)*. This genotype allowed us to measure the spectral sensitivity of the different beetle opsins unhindered by the *Drosophila* visual system (explanation in the text).

### Site-Directed Mutagenesis in *Ch. rajah* Opsins

The 3D structure of *Ch. rajah* UV2 and LW2 opsins were modeled using I-TASSER (Zhang 2008; Yang et al. 2015) under default parameters and viewed in UCSF Chimera (v1.15) (Pettersen et al. 2004). The model template with the highest structural similarity (TM-score) was selected as the squid (*Todarodes pacificus*) rhodopsin crystal structure (PDB model 2Z73A). Beetle opsins aligned to the squid template and the more closely related jumping spider opsin (PDB model 6I9kA) were used to predict binding residues for the bound retinal. We used the protein-ligand binding site prediction program COACH (Yang et al. 2013a, 2013b) and validated these predictions with COACH-D, which reduces steric clashes between protein and ligand (Wu et al. 2018). COACH-D yielded two additional LW sites, 206 and 209, both of which were invariant and not used for further analysis. Predicted binding sites were unaffected by choice of squid or jumping spider template. In addition, we expanded this more conservative method to include all residues that could potentially interact with the chromophore (within 5Å). The best-candidate tuning sites were identified as those with the following characteristics: 1) within the chromophore binding pocket, 2) minimum variation within opsin duplicates and variant between duplicates, 3) previous evidence for spectral shifts in opsins from other organisms, and 4) the structural significance of the amino acids substituted (see Lord et al. 2016).

Four binding sites of interest were chosen: UV2 site 198, UV2 site 285, LW2 site 140, LW2 site 227 (numbered according to *Ch. rajah*). The following mutations were introduced to *Ch. rajah* UV2 and LW2 opsins: UV2 Q198A, UV2 F285Y, LW2 C140T, and LW2 V227C. We followed the QuikChange II Site-Directed Mutagenesis Kit (Agilent) protocol but used 1 min per 500 bp extension time (for primers used see supplementary table S6, Supplementary Material online). Opsins with each mutation were generated as outlined above (single mutants). Additionally, double mutant opsins with both UV or LW mutations were generated using a single mutant as a template (UV2 285 and LW2 140) and introducing the second mutation (UV2 198 and LW2 227) as described above. Single and double mutant sequences were confirmed by Sanger sequencing. Mutated opsin sequences were cloned and transgenic *Drosophila* generated as described above. Fly lines with either one or both mutations were generated for testing. Experimental flies were maintained on cornmeal food at 22 °C with a 12:12 h light cycle. A *C. maculatus* colony was maintained on mung beans at 25 °C with a 12:12 h light cycle.

### Spectral Sensitivity Measurements

The spectral response of transgenic *Drosophila* expressing jewel beetle opsin was tested using ERG and experiments were carried out using similar methods outlined in a previous study (Sharkey et al. 2020). Female *Drosophila* between 3 and 9 days after eclosion were anesthetized on ice and fixed to a metal cone using UV-curing glue (Norland Optical Adhesive NOA68). Borosilicate micropipettes (ID 0.5 mm, OD 1.0 mm, length 10 cm, Sutter, BF100-50-10) were pulled on a Sutter P-2000 laser puller (settings: Heat 350, Fil 4, Vel 50, Del 224, Pul 150). Insect saline (103 mM NaCl, 3 mM KCl, 20 mM BES, 10 mM trehalose, 20 mM sodium bicarbonate, 1 mM sodium phosphate monobasic, 2 mM CaCl_2_ and 4 mM MgCl_2_, all Sigma-Aldrich) was used to fill electrodes. Electroretinogram recordings were made using a blunt electrode placed near the equator of the right eye and a reference electrode was inserted at the median ocellus. Voltage responses were amplified using: MultiClamp 700B amplifier (Molecular devices), EXT-02F (NPI), or Neurolog System (Digitimer). Both stimulus presentation and recording was controlled via a DAQ card (National Instruments) and the software Ephus (Suter et al. 2010). Light was controlled using a monochromator (Cairn Research) with either a 2400 or 1200 line-ruled diffraction grating to provide stimuli between 315 and 550 nm or 450 and 700 nm, respectively. See Sharkey et al. (2020) for full spectral stimulus details.

To determine the stimulus-response (Vlog(I)) function, animals were tested with successively brighter intensities between 1.14 × 10^12^ and 3.60 × 10^16^ photons/cm^2^/s comprising ten 200 ms flashes of light every 10 s. Flies expressing monarch butterfly SW opsin were tested at a lower intensity, between 3.60 × 10^10^ and 6.40 × 10^15^ photons/cm^2^/s due to higher sensitivity to light. Between each test intensity, animals were subject to 100 s of darkness. The responses to the final five flashes of light were used for analysis. The peak wavelength for each Vlog(I) test was dependent on the opsin: 345 nm (UV2), 440 nm (UV1), 500 nm (LW2), or 540 nm (LW1). A sigmoid and Naka-Rushton function were fitted to the data and the intensity of light at half the maximum response was used for spectral tests. Where no saturation of the photoreceptors occurred, the intensity within the linear portion of the curve was used for testing.

For spectral tests, all flies were tested between 315 and 550 nm in steps of 5 nm. LW1 and LW2 flies were also tested between 450 and 700 nm in steps of 5 nm. Wavelengths were divided into three groups from low to high and randomized within. Pulses were presented from one wavelength in each category in the order low to high, yielding a semi-randomized testing procedure, balanced over the wavelength range. Animals were exposed to 200 ms flashes of light every 5 s with ten flashes per wavelength. The responses to the final five flashes of light were used for analysis. The total change in voltage from the baseline before the light flash to the minimum value 10–0 ms before the end of the light flash was used as a measure of photoreceptor response for each ERG. Spectral sensitivity data were smoothed using a Savitzky-Golay filter (data window 15 nm), normalized for each repeat, then an average was taken to give a final curve.

Visual pigment templates were fitted to the sensitivity curves according to equations from Govardovskii et al. (2000) and Stavenga et al. (1993). Templates were better fit to the data using equations from Stavenga et al. (1993) (see supplementary table S1, Supplementary Material online) and were therefore used to estimate *λ*_max_. Six animals were used to test each *C. maculatus* and *Chrysochroa* wild-type opsin and four animals were used to characterize each single and double mutant *Ch. rajah* opsin. Similar experimental procedures were carried out for quantifying *C. maculatus* spectral sensitivity (*n* = 3). The reference electrode was inserted into the head through a small incision into the cuticle and Vlog(I) tests were performed at 500 nm.

## Supplementary Material

msad023_Supplementary_Data

## Data Availability

Raw RNA-seq reads are available at the Sequence Read Archive (BioProject number PRJNA894182). New opsin sequences from this study have been deposited in Genbank (accession numbers OP722923 – OP722954). Spectral sensitivity data and visual pigment templates are provided in the supplementary materials.
