# Supplementary material for: Jewel Beetle Opsin Duplication and Divergence Is the Mechanism for Diverse Spectral Sensitivities
Source: Mol Biol Evol. 2023 Jan 31;40(2):msad023. doi: 10.1093/molbev/msad023 (PMC9937044; doi:10.1093/molbev/msad023)
Supplement: msad023_Supplementary_Data [file msad023_supplementary_data.zip › Supplementary_Material_MBE_Sharkey_final.pdf]

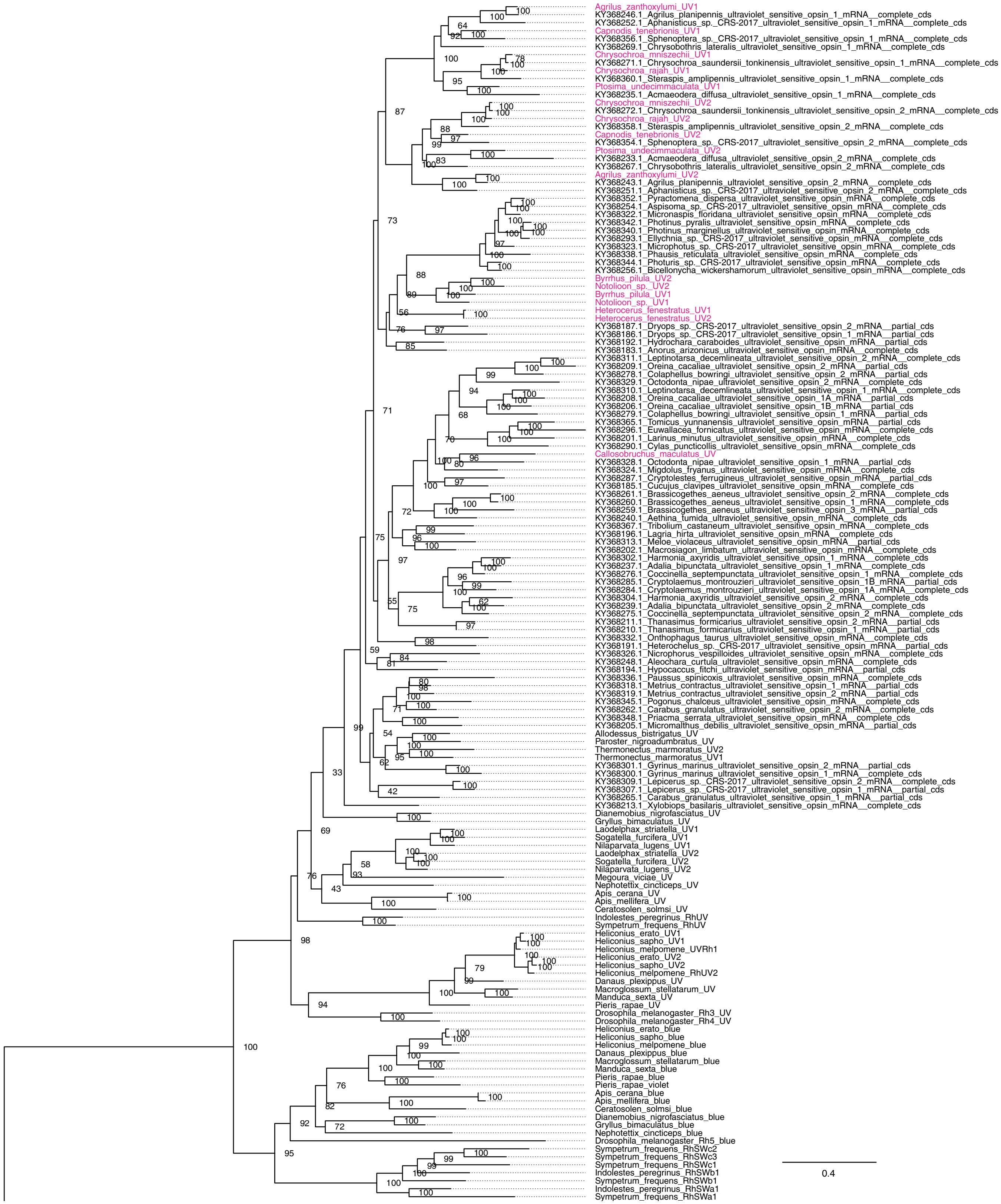
Agrilus_zanthoxylumi_UV1
KY368246.1_Agrilus_planipennis_ultraviolet_sensitive_opsin_1_mRNA__complete_cds
KY368252.1_Aphanisticus_sp._CRS-2017_ultraviolet_sensitive_opsin_1_mRNA__complete_cds
Capnodis_tenebrionis_UV1
KY368356.1_Sphenoptera_sp._CRS-2017_ultraviolet_sensitive_opsin_1_mRNA__complete_cds
KY368269.1_Chrysobothris_lateralis_ultraviolet_sensitive_opsin_1_mRNA__complete_cds
Chrysochroa_mniszechii_UV1
KY368271.1_Chrysochroa_saundersii_tonkinensis_ultraviolet_sensitive_opsin_1_mRNA__complete_cds
Chrysochroa_rajah_UV1
KY368360.1_Steraspis_amplipennis_ultraviolet_sensitive_opsin_1_mRNA__complete_cds
Ptosima_undecimmaculata_UV1
KY368235.1_Acmaeodera_diffusa_ultraviolet_sensitive_opsin_1_mRNA__complete_cds
Chrysochroa_mniszechii_UV2
KY368272.1_Chrysochroa_saundersii_tonkinensis_ultraviolet_sensitive_opsin_2_mRNA__complete_cds
Chrysochroa_rajah_UV2
KY368358.1_Steraspis_amplipennis_ultraviolet_sensitive_opsin_2_mRNA__complete_cds
Capnodis_tenebrionis_UV2
KY368354.1_Sphenoptera_sp._CRS-2017_ultraviolet_sensitive_opsin_2_mRNA__complete_cds
Ptosima_undecimmaculata_UV2
KY368233.1_Acmaeodera_diffusa_ultraviolet_sensitive_opsin_2_mRNA__complete_cds
KY368267.1_Chrysobothris_lateralis_ultraviolet_sensitive_opsin_2_mRNA__complete_cds
Agrilus_zanthoxylumi_UV2
KY368243.1_Agrilus_planipennis_ultraviolet_sensitive_opsin_2_mRNA__complete_cds
KY368251.1_Aphanisticus_sp._CRS-2017_ultraviolet_sensitive_opsin_2_mRNA__complete_cds
KY368352.1_Pyractomena_dispersa_ultraviolet_sensitive_opsin_mRNA__complete_cds
KY368254.1_Aspisoma_sp._CRS-2017_ultraviolet_sensitive_opsin_mRNA__complete_cds
KY368322.1_Micronaspis_floridana_ultraviolet_sensitive_opsin_mRNA__complete_cds
KY368342.1_Photinus_pyralis_ultraviolet_sensitive_opsin_mRNA__complete_cds
KY368340.1_Photinus_marginellus_ultraviolet_sensitive_opsin_mRNA__complete_cds
KY368293.1_Ellychnia_sp._CRS-2017_ultraviolet_sensitive_opsin_mRNA__complete_cds
KY368323.1_Microphotus_sp._CRS-2017_ultraviolet_sensitive_opsin_mRNA__complete_cds
KY368338.1_Phausis_reticulata_ultraviolet_sensitive_opsin_mRNA__complete_cds
KY368344.1_Photuris_sp._CRS-2017_ultraviolet_sensitive_opsin_mRNA__complete_cds
KY368256.1_Bicellonycha_wickershamorum_ultraviolet_sensitive_opsin_mRNA__complete_cds
Byrrhus_pilula_UV2
Notolioon_sp._UV2
Byrrhus_pilula_UV1
Notolioon_sp._UV1
Heterocerus_fenestratus_UV1
Heterocerus_fenestratus_UV2
KY368187.1_Dryops_sp._CRS-2017_ultraviolet_sensitive_opsin_2_mRNA__partial_cds
KY368186.1_Dryops_sp._CRS-2017_ultraviolet_sensitive_opsin_1_mRNA__partial_cds
KY368192.1_Hydrochara_caraboides_ultraviolet_sensitive_opsin_mRNA__partial_cds
KY368183.1_Anorus_arizonicus_ultraviolet_sensitive_opsin_mRNA__complete_cds
KY368311.1_Leptinotarsa_decemlineata_ultraviolet_sensitive_opsin_2_mRNA__complete_cds
KY368209.1_Oreina_cacaliae_ultraviolet_sensitive_opsin_2_mRNA__partial_cds
KY368278.1_Colaphellus_bowringi_ultraviolet_sensitive_opsin_2_mRNA__partial_cds
KY368329.1_Octodonta_nipae_ultraviolet_sensitive_opsin_2_mRNA__complete_cds
KY368310.1_Leptinotarsa_decemlineata_ultraviolet_sensitive_opsin_1_mRNA__complete_cds
KY368208.1_Oreina_cacaliae_ultraviolet_sensitive_opsin_1A_mRNA__partial_cds
KY368206.1_Oreina_cacaliae_ultraviolet_sensitive_opsin_1B_mRNA__partial_cds
KY368279.1_Colaphellus_bowringi_ultraviolet_sensitive_opsin_1_mRNA__partial_cds
KY368365.1_Tomicus_yunnanensis_ultraviolet_sensitive_opsin_mRNA__partial_cds
KY368296.1_Euwallacea_fornicatus_ultraviolet_sensitive_opsin_mRNA__complete_cds
KY368201.1_Larinus_minutus_ultraviolet_sensitive_opsin_mRNA__complete_cds
KY368290.1_Cylas_puncticollis_ultraviolet_sensitive_opsin_mRNA__complete_cds
Callosobruchus_maculatus_UV
KY368328.1_Octodonta_nipae_ultraviolet_sensitive_opsin_1_mRNA__partial_cds
KY368324.1_Migdolus_fryanus_ultraviolet_sensitive_opsin_mRNA__complete_cds
KY368287.1_Cryptolestes_ferrugineus_ultraviolet_sensitive_opsin_mRNA__partial_cds
KY368185.1_Cucujus_clavipes_ultraviolet_sensitive_opsin_mRNA__complete_cds
KY368261.1_Brassicogethes_aeneus_ultraviolet_sensitive_opsin_2_mRNA__complete_cds
KY368260.1_Brassicogethes_aeneus_ultraviolet_sensitive_opsin_1_mRNA__complete_cds
KY368259.1_Brassicogethes_aeneus_ultraviolet_sensitive_opsin_3_mRNA__partial_cds
KY368240.1_Aethina_tumida_ultraviolet_sensitive_opsin_mRNA__complete_cds
KY368367.1_Tribolium_castaneum_ultraviolet_sensitive_opsin_mRNA__complete_cds
KY368196.1_Lagria_hirta_ultraviolet_sensitive_opsin_mRNA__complete_cds
KY368313.1_Meloe_violaceus_ultraviolet_sensitive_opsin_mRNA__partial_cds
KY368202.1_Macrosiagon_limbatum_ultraviolet_sensitive_opsin_mRNA__complete_cds
KY368302.1_Harmonia_axyridis_ultraviolet_sensitive_opsin_1_mRNA__complete_cds
KY368237.1_Adalia_bipunctata_ultraviolet_sensitive_opsin_1_mRNA__complete_cds
KY368276.1_Coccinella_septempunctata_ultraviolet_sensitive_opsin_1_mRNA__complete_cds
KY368285.1_Cryptolaemus_montrouzieri_ultraviolet_sensitive_opsin_1B_mRNA__partial_cds
KY368284.1_Cryptolaemus_montrouzieri_ultraviolet_sensitive_opsin_1A_mRNA__complete_cds
KY368304.1_Harmonia_axyridis_ultraviolet_sensitive_opsin_2_mRNA__complete_cds
KY368239.1_Adalia_bipunctata_ultraviolet_sensitive_opsin_2_mRNA__complete_cds
KY368275.1_Coccinella_septempunctata_ultraviolet_sensitive_opsin_2_mRNA__complete_cds
KY368211.1_Thanasimus_formicarius_ultraviolet_sensitive_opsin_2_mRNA__partial_cds
KY368210.1_Thanasimus_formicarius_ultraviolet_sensitive_opsin_1_mRNA__partial_cds
KY368332.1_Onthophagus_taurus_ultraviolet_sensitive_opsin_mRNA__complete_cds
KY368191.1_Heterochelus_sp._CRS-2017_ultraviolet_sensitive_opsin_mRNA__partial_cds
KY368326.1_Nicrophorus_vespilloides_ultraviolet_sensitive_opsin_mRNA__complete_cds
KY368248.1_Aleochara_curtula_ultraviolet_sensitive_opsin_mRNA__complete_cds
KY368194.1_Hypocaccus_fitchi_ultraviolet_sensitive_opsin_mRNA__partial_cds
KY368336.1_Paussus_spinicoxis_ultraviolet_sensitive_opsin_mRNA__complete_cds
KY368318.1_Metrius_contractus_ultraviolet_sensitive_opsin_1_mRNA__partial_cds
KY368319.1_Metrius_contractus_ultraviolet_sensitive_opsin_2_mRNA__partial_cds
KY368345.1_Pogonus_chalceus_ultraviolet_sensitive_opsin_mRNA__complete_cds
KY368262.1_Carabus_granulatus_ultraviolet_sensitive_opsin_2_mRNA__complete_cds
KY368348.1_Priacma_serrata_ultraviolet_sensitive_opsin_mRNA__complete_cds
KY368205.1_Micromalthus_debilis_ultraviolet_sensitive_opsin_mRNA__partial_cds
Allodessus_bistrigatus_UV
Paroster_nigroadumbratus_UV
Thermonectus_marmoratus_UV2
Thermonectus_marmoratus_UV1
KY368301.1_Gyrinus_marinus_ultraviolet_sensitive_opsin_2_mRNA__partial_cds
KY368300.1_Gyrinus_marinus_ultraviolet_sensitive_opsin_1_mRNA__complete_cds
KY368309.1_Lepicerus_sp._CRS-2017_ultraviolet_sensitive_opsin_2_mRNA__complete_cds
KY368307.1_Lepicerus_sp._CRS-2017_ultraviolet_sensitive_opsin_1_mRNA__partial_cds
KY368265.1_Carabus_granulatus_ultraviolet_sensitive_opsin_1_mRNA__partial_cds
KY368213.1_Xylobiops_basilaris_ultraviolet_sensitive_opsin_mRNA__complete_cds
Dianemobius_nigrofasciatus_UV
Gryllus_bimaculatus_UV
Laodelphax_striatella_UV1
Sogatella_furcifera_UV1
Nilaparvata_lugens_UV1
Laodelphax_striatella_UV2
Sogatella_furcifera_UV2
Nilaparvata_lugens_UV2
Megoura_viciae_UV
Nephotettix_cincticeps_UV
Apis_cerana_UV
Apis_mellifera_UV
Ceratosolen_solmsi_UV
Indolestes_peregrinus_RhUV
Sympetrum_frequens_RhUV
Heliconius_erato_UV1
Heliconius_sapho_UV1
Heliconius_melpomene_UVRh1
Heliconius_erato_UV2
Heliconius_sapho_UV2
Heliconius_melpomene_RhUV2
Danaus_plexippus_UV
Macroglossum_stellatarum_UV
Manduca_sexta_UV
Pieris_rapae_UV
Drosophila_melanogaster_Rh3_UV
Drosophila_melanogaster_Rh4_UV
Heliconius_erato_blue
Heliconius_sapho_blue
Heliconius_melpomene_blue
Danaus_plexippus_blue
Macroglossum_stellatarum_blue
Manduca_sexta_blue
Pieris_rapae_blue
Pieris_rapae_violet
Apis_cerana_blue
Apis_mellifera_blue
Ceratosolen_solmsi_blue
Dianemobius_nigrofasciatus_blue
Gryllus_bimaculatus_blue
Nephotettix_cincticeps_blue
Drosophila_melanogaster_Rh5_blue
Sympetrum_frequens_RhSWc2
Sympetrum_frequens_RhSWc3
Sympetrum_frequens_RhSWc1
Indolestes_peregrinus_RhSWb1
Sympetrum_frequens_RhSWb1
Indolestes_peregrinus_RhSWa1
Sympetrum_frequens_RhSWa1
0.4

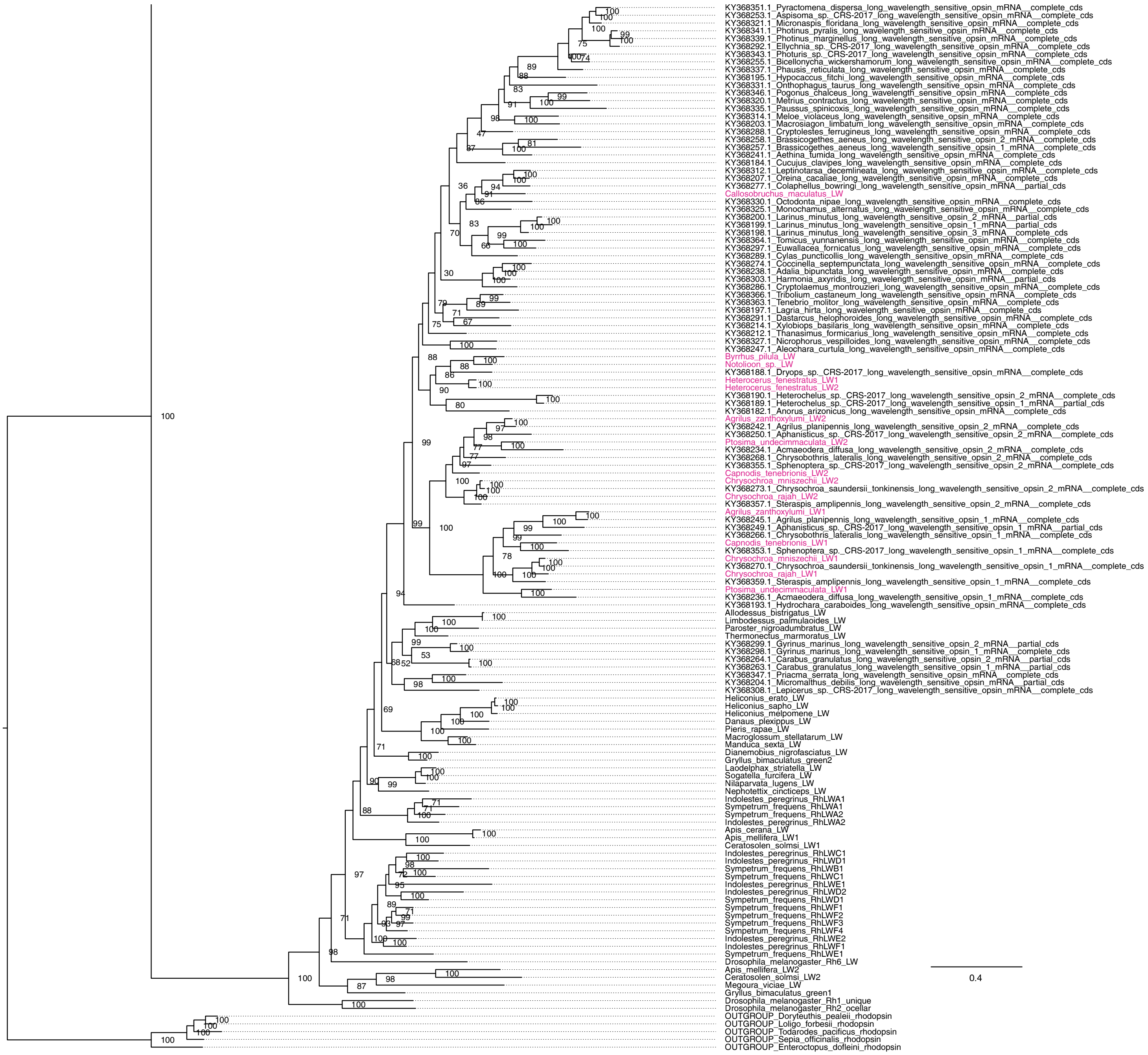

Fig. S1.

Maximum likelihood opsin phylogeny with new sequences from this study (pink) and additional insect opsins (black), focusing on beetle opsins. Node values represent UFboot support values and are based on 10,000 replicates.

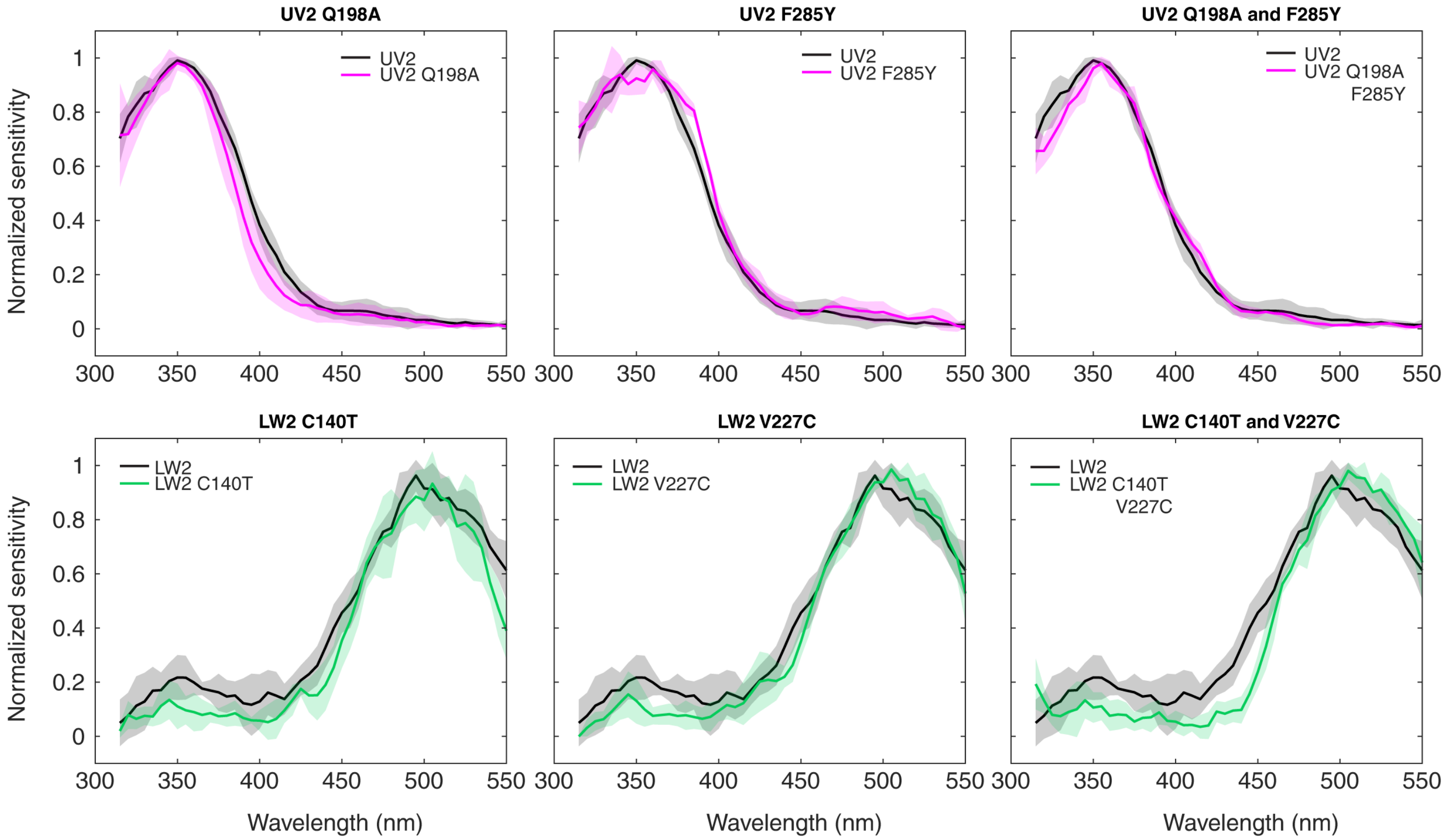

Fig. S2.

Spectral sensitivity curves of *Drosophila* expressing mutant *Chrysochroa rajah* opsin (n = 4) alongside the wildtype UV2 or LW2 opsin (n = 6). Error shown is standard deviation.

Table S1.
Curve fitting statistics and λ_{max} for each opsin and six opsin mutants fitted with Govardovskii *et al.* (2000) (GOV) and Stavenga *et al.* (1993) (SSH) visual pigment templates. Photopigment sensitivity was tested either between 315 - 550 or 450 - 700 nm and in the case of LW2, curves were fitted to both datasets.

Opsin	Experimental range (nm)	Species	Wavelength at peak response (nm)	GOV template λmax (nm)	GOV adj R^2	SSH template λmax (nm)	SSH adj R^2
UV1	315 - 550	*Chrysochroa mniszechii*	460	443	-0.853	442	-0.683
UV2	315 - 550	*Chrysochroa mniszechii*	355	361	0.905	357	0.939
LW1	450 - 700	*Chrysochroa mniszechii*	565	571	0.875	572	0.878
LW2	315 - 550	*Chrysochroa mniszechii*	495	506	0.923	507	0.940
LW2	450 - 700	*Chrysochroa mniszechii*	505	515	0.934	515	0.931
UV1	315 - 550	*Chrysochroa rajah*	420	432	0.082	431	0.194
UV2	315 - 550	*Chrysochroa rajah*	350	360	0.914	356	0.950
LW1	450 - 700	*Chrysochroa rajah*	595	584	0.970	584	0.959
LW2	315 - 550	*Chrysochroa rajah*	495	509	0.949	509	0.956
LW2	450 - 700	*Chrysochroa rajah*	510	516	0.922	516	0.918
UV	315 - 550	*Callosobruchus maculatus*	360	367	0.877	363	0.917
LW	315 - 550	*Callosobruchus maculatus*	505	511	0.954	511	0.965
UV2 Q198A	315 - 550	*Chrysochroa rajah*	350	356	0.955	352	0.979
UV2 F285Y	315 - 550	*Chrysochroa rajah*	360	363	0.869	359	0.907
LW2 C140T	315 - 550	*Chrysochroa rajah*	505	507	0.947	507	0.942
LW2 V227C	315 - 550	*Chrysochroa rajah*	505	510	0.970	511	0.973
UV2 Q198A F285Y	315 - 550	*Chrysochroa rajah*	355	362	0.919	358	0.957
LW2 C140T V227C	315 - 550	*Chrysochroa rajah*	505	519	0.949	519	0.936
SW	315 - 550	*Danaus plexippus*	455	447	0.674	447	0.735

Table S2.
Buprestid opsin residues predicted to be within the chromophore binding pocket, based on 3D protein modelling of UV2 *Chrysochroa rajah* opsin. Sites are numbered according to bovine rhodopsin and *C. rajah* UV2. Rows are colored according to *Chrysochroa* spectral sensitivity. Sites used for point mutation are in bold.

Species	Opsin	Predicted chromophore binding sites																	
Bos taurus	Rh	113	117	118	121	122	**186**	187	188	189	191	207	212	**261**	265	268	269	292	296
		E	A	T	G	E	**S**	C	G	I	Y	M	F	**F**	W	Y	A	A	K
Chrysochroa rajah	UV2	125	129	130	133	134	**198**	199	200	201	203	217	222	**285**	289	292	293	316	320
Acmaeodera diffusa	UV2	F	G	T	G	I	**Q**	C	G	I	Y	L	Y	**F**	W	Y	G	A	K
Agrilus planipennis	UV2	F	G	T	G	I	**Q**	C	T	F	Y	L	Y	**F**	W	Y	A	A	K
Agrilus zanthoxylumi	UV2	F	G	T	G	I	**Q**	C	S	F	Y	L	Y	**F**	W	Y	A	A	K
Aphanisticus sp.	UV2	F	G	T	G	I	**Q**	C	T	F	Y	L	Y	**F**	W	Y	A	A	K
Capnodis tenebrionis	UV2	F	G	T	G	I	**Q**	C	T	F	Y	L	Y	**F**	W	Y	A	A	K
Chrysobothris lateralis	UV2	F	G	T	G	I	**Q**	C	T	F	Y	L	Y	**F**	W	Y	A	A	K
Chrysochroa mniszechii	UV2	F	G	T	G	I	**Q**	C	S	F	Y	L	Y	**F**	W	Y	A	A	K
Chrysochroa tonkinensis	UV2	F	G	T	G	I	**Q**	C	T	F	Y	L	Y	**F**	W	Y	A	A	K
Chrysochroa rajah	UV2	F	G	T	G	I	**Q**	C	T	F	Y	L	Y	**F**	W	Y	A	A	K
Ptosima undecimmaculata	UV2	F	G	S	G	I	**Q**	C	G	V	Y	L	Y	**F**	W	Y	A	A	K
Sphenoptera sp.	UV2	F	G	T	G	I	**Q**	C	S	F	Y	L	Y	**F**	W	Y	A	A	K
Steraspis amplipennis	UV2	F	G	T	G	I	**Q**	C	S	F	Y	L	Y	**F**	W	Y	A	A	K
Acmaeodera diffusa	UV1	F	G	T	G	I	**T**	C	S	F	Y	I	Y	**F**	W	Y	A	A	K
Agrilus planipennis	UV1	F	G	S	G	I	**A**	C	S	F	Y	I	Y	**Y**	W	Y	A	A	K
Agrilus zanthoxylumi	UV1	F	G	S	G	I	**A**	C	S	F	Y	I	Y	**Y**	W	Y	A	A	K
Aphanisticus sp.	UV1	F	G	S	G	I	**A**	C	S	F	Y	I	Y	**Y**	W	Y	A	A	K
Capnodis tenebrionis	UV1	F	G	S	G	I	**A**	C	S	F	Y	I	Y	**Y**	W	Y	A	A	K
Chrysobothris lateralis	UV1	F	G	S	G	I	**A**	C	S	F	Y	I	Y	**Y**	W	Y	A	A	K
Chrysochroa mniszechii	UV1	F	G	S	G	I	**A**	C	S	F	Y	I	Y	**Y**	W	Y	A	S	K
Chrysochroa tonkinensis	UV1	F	G	S	G	I	**A**	C	S	F	Y	I	Y	**Y**	W	Y	A	S	K
Chrysochroa rajah	UV1	F	G	S	G	I	**A**	C	S	F	Y	I	Y	**Y**	W	Y	A	S	K
Ptosima undecimmaculata	UV1	Y	G	S	G	I	**A**	C	S	F	Y	I	Y	**F**	W	Y	A	A	K
Sphenoptera sp.	UV1	F	G	S	G	I	**A**	C	T	F	Y	I	Y	**Y**	W	Y	A	A	K
Steraspis amplipennis	UV1	F	G	T	G	I	**A**	C	S	F	Y	I	Y	**F**	W	Y	A	A	K

Table S3.

Buprestid opsin residues predicted to be within the chromophore binding pocket, based on 3D protein modelling of LW2 *Chrysochroa rajah* opsin. Sites are numbered according to bovine rhodopsin and *C. rajah* LW2. Rows are colored according to *Chrysochroa* spectral sensitivity. Sites used for point mutation are in bold.

Species	Opsin	Predicted chromophore binding sites																
Bos taurus	Rh	113	117	118	121	**122**	186	187	189	207	**211**	212	261	265	268	269	292	296
		E	A	T	G	**E**	S	C	I	M	**H**	F	F	W	Y	A	A	K
Chrysochroa rajah	LW2	131	135	136	139	**140**	204	205	207	223	**227**	228	290	294	297	298	320	324
Acmaeodera diffusa	LW2	H	G	S	G	**C**	A	C	T	Y	**V**	Y	W	W	Y	L	S	K
Agrilus planipennis	LW2	Y	G	S	G	**C**	A	C	T	Y	**V**	Y	W	W	Y	L	S	K
Agrilus zanthoxylumi	LW2	Y	G	S	G	**C**	A	C	T	Y	**V**	Y	W	W	Y	L	S	K
Aphanisticus sp.	LW2	Y	G	S	G	**C**	A	C	T	Y	**V**	Y	W	W	Y	L	S	K
Capnodis tenebrionis	LW2	Y	G	S	G	**C**	A	C	T	Y	**V**	Y	W	W	Y	L	S	K
Chrysobothris lateralis	LW2	Y	G	S	G	**C**	A	C	T	Y	**C**	Y	W	W	Y	L	S	K
Chrysochroa mniszechii	LW2	Y	G	S	G	**C**	A	C	T	Y	**V**	Y	W	W	Y	L	S	K
Chrysochroa tonkinensis	LW2	Y	G	S	G	**C**	A	C	T	Y	**V**	Y	W	W	Y	L	S	K
Chrysochroa rajah	LW2	Y	G	S	G	**C**	S	C	T	Y	**V**	Y	W	W	Y	L	S	K
Ptosima undecimmaculata	LW2	Y	G	S	G	**C**	A	C	T	Y	**V**	Y	W	W	Y	L	S	K
Sphenoptera sp.	LW2	Y	G	S	G	**C**	A	C	T	Y	**V**	Y	W	W	Y	L	S	K
Steraspis amplipennis	LW2	Y	G	S	G	**C**	A	C	T	Y	**V**	Y	W	W	Y	L	S	K
Acmaeodera diffusa	LW1	Y	G	S	G	**T**	A	C	T	Y	**C**	Y	W	W	Y	L	S	K
Agrilus planipennis	LW1	Y	G	S	G	**C**	A	C	T	Y	**C**	Y	W	W	Y	L	S	K
Agrilus zanthoxylumi	LW1	Y	G	S	G	**C**	A	C	T	Y	**C**	Y	W	W	Y	L	S	K
Aphanisticus sp.	LW1	Y	G	S	G	**C**	A	C	T	Y	**C**	Y	W	W	Y	L	S	K
Capnodis tenebrionis	LW1	Y	G	S	G	**C**	A	C	T	Y	**C**	Y	W	W	Y	L	S	K
Chrysobothris lateralis	LW1	Y	G	S	G	**C**	A	C	T	Y	**C**	Y	W	W	Y	L	S	K
Chrysochroa mniszechii	LW1	Y	G	S	G	**T**	A	C	T	Y	**C**	Y	W	W	Y	A	S	K
Chrysochroa tonkinensis	LW1	Y	G	S	G	**T**	A	C	T	Y	**C**	Y	W	W	Y	A	S	K
Chrysochroa rajah	LW1	Y	G	S	G	**T**	A	C	T	Y	**C**	Y	W	W	Y	T	S	K
Ptosima undecimmaculata	LW1	Y	G	S	G	**T**	A	C	T	Y	**C**	Y	W	W	Y	L	S	K
Sphenoptera sp.	LW1	Y	G	S	G	**C**	A	C	T	Y	**C**	Y	W	W	Y	L	S	K
Steraspis amplipennis	LW1	Y	G	S	G	**T**	A	C	T	Y	**C**	Y	W	W	Y	M	S	K

Table S4.
Sample information for RNA-seq data used in this study. All RNA-seq data were paired-end reads.

Species	Tissue	Sex	Family	Subfamily	Data type	Sequencing platform	SRA code/reference
Acmaeodera diffusa	-	Male + female	Buprestidae	Polycestinae	Opsins	-	(Lord *et al.,* 2016)
Agrilus planipennis	-	Male + female	Buprestidae	Agrilinae	Opsins	-	(Lord *et al.,* 2016)
Agrilus zanthoxylumi	Head	Male	Buprestidae	Agrilinae	RNA-seq	Illumina NovaSeq 6000	SRR10867870
Aphanisticus sp.	-	-	Buprestidae	Agrilinae	Opsins	-	(Lord *et al.,* 2016)
Capnodis tenebrionis	Whole body without antennae	Male	Buprestidae	Chrysochroinae	RNA-seq	Illumina HiSeq 2500	ERR3039978
Capnodis tenebrionis	Whole body without antennae	Female	Buprestidae	Chrysochroinae	RNA-seq	Illumina HiSeq 2500	ERR3039976
Chrysobothris lateralis	-	Male + female	Buprestidae	Buprestinae	Opsins	-	(Lord *et al.,* 2016)
Chrysochroa rajah	Head	Female	Buprestidae	Chrysochroinae	RNA-seq	Illumina HiSeq 2000	This study
Chrysochroa tonkinensis	-	Male	Buprestidae	Chrysochroinae	Opsins	-	(Lord *et al.,* 2016)
Chrysochroa mniszechii	Head	Male	Buprestidae	Chrysochroinae	RNA-seq	Illumina HiSeq 2000	This study
Ptosima undecimmaculata	Whole body	-	Buprestidae	Polycestinae	RNA-seq	Illumina HiSeq 2000	SRR2083717
Sphenoptera sp.	-	Female	Buprestidae	Chrysochroinae	Opsins	-	(Lord *et al.,* 2016)
Steraspis amplipennis	-	Female	Buprestidae	Chrysochroinae	Opsins	-	(Lord *et al.,* 2016)
Byrrhus pilula	Whole body	-	Byrrhidae	-	RNA-seq	Illumina HiSeq 2000	SRR2083638
Notolioon sp.	Whole body	-	Byrrhidae	-	RNA-seq	Illumina HiSeq 2000	SRR2083702
Callosobruchus maculatus	Whole body	Male	Chrysomelidae	Bruchinae	RNA-seq	Illumina HiSeq 2000	SRR3113341<br>SRR3113361<br>SRR3113418
Callosobruchus maculatus	Whole body	Female	Chrysomelidae	Bruchinae	RNA-seq	Illumina HiSeq 2000	SRR3113380<br>SRR3113382<br>SRR3113383
Heterocerus fenestratus	Whole body	-	Heteroceridae	-	RNA-seq	Illumina HiSeq 2000	SRR2083670

Table S5.
Primers used to amplify beetle opsins used in this study and the monarch butterfly, *Danaus plexippus*, including plasmid overhang (black), bovine epitope (blue) and opsin (red).

Opsin	Species	Primer direction	Sequence
UV1	*Chrysochroa rajah*	F	CCAAAACACACCTAGGCCACCATGAAAGAAATGATGTTCAA
UV1	*Chrysochroa rajah*	R	CGCGGCCGCTCCTAGGTTAGGCAGGCGCCACCTGGCTGGTCTCTGTGGTTCTCGTAGC CGGAT
UV2	*Chrysochroa rajah*	F	CCAAAACACACCTAGGCCACCATGCATCTGACAAATTATAG
UV2	*Chrysochroa rajah*	R	CGCGGCCGCTCCTAGGCTAGGCAGGCGCCACCTGGCTGGTCTCTGTAGGCTTGTTT GTAGAAG
LW1	*Chrysochroa rajah*	F	CCAAAACACACCTAGGCCACCATGTCCATTGTGGGAGAACC
LW1	*Chrysochroa rajah*	R	CGCGGCCGCTCCTAGGTCAGGCAGGCGCCACCTGGCTGGTCTCTGTCGCAGGTGTCG TCTTTG
LW2	*Chrysochroa rajah*	F	CCAAAACACACCTAGGCCACCATGTCGGCTTTGGGCGAACC
LW2	*Chrysochroa rajah*	R	CGCGGCCGCTCCTAGGCTAGGCAGGCGCCACCTGGCTGGTCTCTGTCGCCGCAACT TTTTCTT
UV1	*Chrysochroa mniszechii*	F	CCAAAACACACCTAGGCCACCATGAAAGAAATAATGTTCAA
UV1	*Chrysochroa mniszechii*	R	CGCGGCCGCTCCTAGGTTAGGCAGGCGCCACCTGGCTGGTCTCTGTGGTTCTCGTA GCCGCAT
UV2	*Chrysochroa mniszechii*	F	CCAAAACACACCTAGGCCACCATGCATCTGACAAATTATAG
UV2	*Chrysochroa mniszechii*	R	CGCGGCCGCTCCTAGGCTAGGCAGGCGCCACCTGGCTGGTCTCTGTAGGCTTGTTT GTAGAAG
LW1	*Chrysochroa mniszechii*	F	CCAAAACACACCTAGGCCACCATGTCCATAGTGGGAGAACC
LW1	*Chrysochroa mniszechii*	R	CGCGGCCGCTCCTAGGTCAGGCAGGCGCCACCTGGCTGGTCTCTGTCGCAGACGCC GTCTTTC
LW2	*Chrysochroa mniszechii*	F	CCAAAACACACCTAGGCCACCATGTCGGCTTTGGGCGAACC
LW2	*Chrysochroa mniszechii*	R	CGCGGCCGCTCCTAGGCTAGGCAGGCGCCACCTGGCTGGTCTCTGTCGCCGCAACT TTTTCTT
UV	*Callosobruchus maculatus*	F	CCAAAACACACCTAGGCCACCATGAGATTCGAAAACTGGAC
UV	*Callosobruchus maculatus*	R	CGCGGCCGCTCCTAGGCTAGGCAGGCGCCACCTGGCTGGTCTCTGTAGTTGTGGCC GTTGAAT
LW	*Callosobruchus maculatus*	F	CCAAAACACACCTAGGCCACCATGTCGGTCATGGGAGAGCC
LW	*Callosobruchus maculatus*	R	CGCGGCCGCTCCTAGGTTAGGCAGGCGCCACCTGGCTGGTCTCTGTGGCATTTGATT TTTCGT
SW	*Danaus plexippus*	F	CCAAAACACACCTAGGCCACCATGGCGACAAACTTCACAGA
SW	*Danaus plexippus*	R	CGCGGCCGCTCCTAGGTCAGGCAGGCGCCACCTGGCTGGTCTCTGTCGCTTCAGCA GGATTCT

Table S6.
Primers used to introduce point mutations (shown in red) in UV2 and LW2 *C. rajah* opsins.

Opsin	Amino substitution	Nucleotide substitutions	Primer direction	Primer sequence
UV2	Q198A	C592G, A593C	F	GTTCCAGAGGGATTTTTGACGGCATGTACGTTTGATTACTTGAC
UV2	Q198A	C592G, A593C	R	GTCAAGTAATCAAACGTACATGCCGTCAAAAATCCCTCTGGAAC
UV2	F285Y	T854A	F	AAAAGTAGCAATAACTATCTGTTTCTTGTATGTCGCATCATGGAC
UV2	F285Y	T854A	R	GTCCATGATGCGACATACAAGAAACAGATAGTTATTGCTACTTTT
LW2	C140T	T418A, G419C	F	GTATGTTTGGTTCCCTCTTTGGAACTACTTCTATTTGGACAATGACTG
LW2	C140T	T418A, G419C	R	CAGTCATTGTCCAAATAGAAGTAGTTCCAAAGAGGGAACCAAACATAC
LW2	V227C	G679T, T680G	F	CATCTTTGCGTACTCAGCCTTTTGCTATTTTCTGCCTCTTAGCATC
LW2	V227C	G679T, T680G	R	GATGCTAAGAGGCAGAAAATAGCAAAAGGCTGAGTACGCAAAGATG
